# Comparison of Endoscope-Assisted and Microscope-Assisted Tubular Surgery for Lumbar Laminectomies and Discectomies: Minimum 2-Year Follow-Up Results

**DOI:** 10.1155/2019/5321580

**Published:** 2019-04-24

**Authors:** Yaqing Zhang, Fanli Chong, Chencheng Feng, Yan Wang, Yue Zhou, Bo Huang

**Affiliations:** Department of Orthopedics, Xinqiao Hospital, The Third Military Medical University, Chongqing 400037, China

## Abstract

**Purpose:**

This study aimed to evaluate the clinical outcomes of endoscope-assisted and microscope-assisted tubular surgery for lumbar laminectomies and discectomies.

**Methods:**

Three hundred and seven patients with lumbar spinal stenosis (LSS) or lumbar disc herniation (LDH) at L3–4, L4–5, and L5-S1 were included in this study. The patients were treated with endoscope-assisted or microscope-assisted tubular surgery. Data on patient demographic characteristics and operative results, including ages, blood loss, operative times, hospital stay, and surgical complications were collected. Clinical outcomes were assessed based on pre- and postoperative Visual Analog Scale (VAS) scores for low-back pain (LBP) and leg pain, Oswestry Disability Index (ODI), and Japanese Orthopaedic Association (JOA) scale.

**Results:**

Both tubular-based endoscope-assisted and microscope-assisted surgery were effective in relieving acute radicular symptoms. The results showed characteristic differences in operating times between endoscope-assisted and microscope-assisted procedures and between discectomies and laminectomies. At the last follow-up, VAS scores of LBP and leg pain, JOA scores, and ODI scores were significantly better than preoperative correlates in all groups. There were no differences between endoscope-assisted and microscope-assisted discectomies for LDH in JOA scores, ODI scores, and VAS scores, while the microscope-assisted laminectomies related to better JOA recovery rate for LSS.

**Conclusions:**

Endoscope-assisted and microscope-assisted tubular discectomies resulted in similar clinical outcomes for LDH, while the microscope-assisted surgery may relate to better recovery rate for LSS, less surgical time, and less intraoperative dural tear.

## 1. Introduction

Degenerative changes of the lumbar spine such as spinal stenosis and disc herniation constitute common cause of back pain and radiculopathy. Surgical treatment is offered to patients that is refractory to conservative treatment [1, 2], and surgical options include open laminectomy and discectomy, microdiscectomy (MD), and microendoscopic discectomy (MED). The first successful open laminectomy and discectomy was described in 1934 [3]. With the introduction of the operative microscope in the 1970s, microsurgical discectomy technique has become the gold standard of treatment for LDH and widely accepted by surgeons [4, 5]. A tubular endoscopic approach, MED, was first described in 1997 as a minimally invasive alternative to open surgical techniques [6, 7]. In recent years, rapid technological advancement led to the increasingly popular tubular retractors in conjunction with the microscope or endoscope for the treatment of degenerative lumbar disease [8–12]. Although many studies have compared the clinical outcomes between conventional MD and tubular MED [13–15], or open MD and tubular MD [16, 17], to our knowledge, there have been no reports with regard to endoscope-assisted and microscope-assisted laminectomy and discectomy with similar tubular retractors for symptomatic LSS and LDH. Is the difference only the way of visualization between MED and MD? In this study, we compared and assessed retrospectively 2-year follow-up results of endoscope-assisted or microscope-assisted tubular laminectomy or discectomy in patients with LSS or LDH.

## 2. Material and Methods

### 2.1. Patients

This study involves a retrospectively analysis of 307 patients who underwent endoscope-assisted or microscope-assisted tubular laminectomy or discectomy between June 2014 and January 2016. All patients were refractory to conservative treatment. Patients with spondylolisthesis, cauda equina syndrome, previous spinal surgery at the same disk level, or less distinct nerve root compression on magnetic resonance imaging (MRI) have been excluded.

The patients were divided into four groups as follows: group A consists of 35 patients (19 men and 16 women) with LSS treated with endoscope-assisted tubular surgery for laminectomies; group B consists of 30 patients (16 men and 14 women) with LSS treated with microscope-assisted tubular surgery for laminectomies; group C consists of 127 patients (74 men and 53 women) with LDH treated with endoscope-assisted tubular surgery for discectomy; group D consists of 115 patients (63 men and 52 women) with LDH treated with microscope-assisted tubular surgery for discectomy (Table 1).

The data of 307 patients with a minimum of two years of follow-up were collected and reviewed. All patients were examined and questioned by an independent researcher. The patient outcomes were scored based on operative times, intraoperative blood loss, pre- and postoperative VAS for LBP and leg pain, and ODI and JOA scale at follow-up appointments 12, 52, and 104 months after surgery. The JOA recovery rate was calculated according to the following formula: (104-week follow-up score - preoperative JOA score) / (29 - preoperative JOA score) × 100%. The complication was assessed including cerebrospinal fluid (CSF) leak rate, neurological injury, infection rate, and number of reoperations by retrospectively reviewing patient charts. Clinical outcomes were done over the phone if they could not be obtained from a follow-up visit, while 47 patients refused the ODI and JOA questionnaire via phone at 12- and 52-week time points. The data of ODI and JOA score at follow-up time points (12 and 52 months) were excluded. The study was approved by the medical ethics committee of the hospital, and written informed consent was obtained from all patients.

### 2.2. Surgical Technique

**Figure 1:**
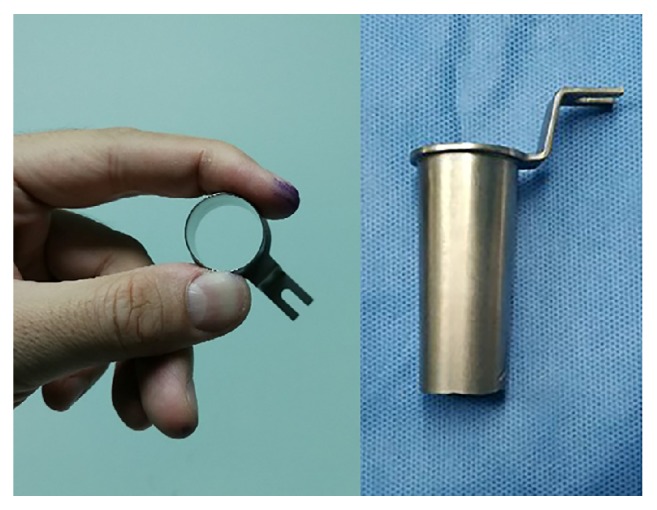
Zista tapered retractor is 22 mm in diameter on the upper end and tapers to a diameter of 18 mm at the opposite end.

**Figure 2:**
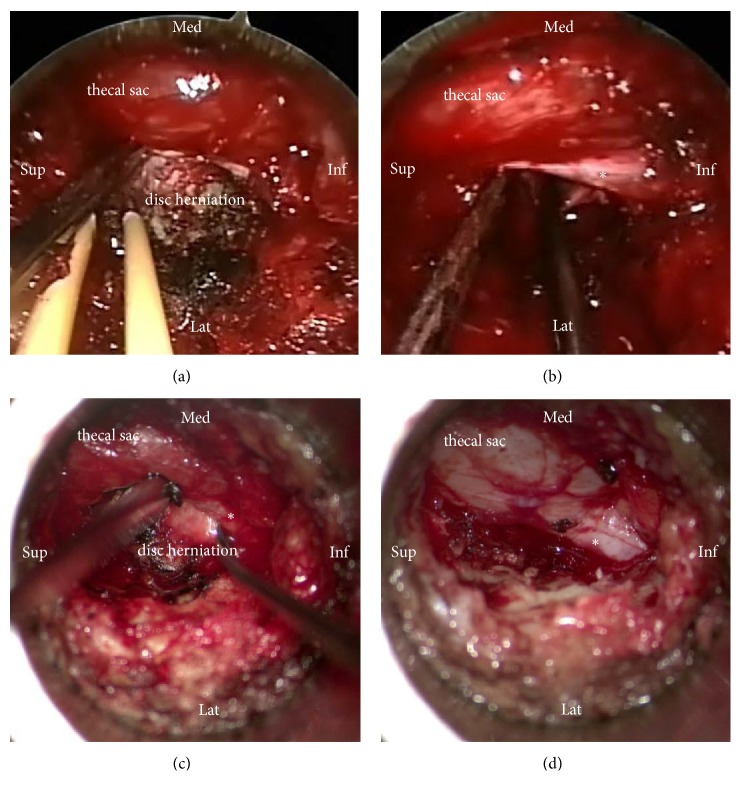
Intraoperative images of endoscope-assisted and microscope-assisted tubular surgery. The thecal sac and the disc herniation are visualized in endoscope-assisted surgery (a). After discectomy, the nerve root (asterisk) is free of compression in endoscope-assisted surgery (b). The thecal sac and the disc herniation are visualized in microscope-assisted surgery (c). After discectomy, the nerve root (asterisk) is free of compression in microscope-assisted surgery (d).

Participating surgeons performed both types of surgical techniques and had broad experience in both techniques. The patient was placed in a prone position under general anesthesia. The operative level was verified fluoroscopically and a paramedian skin incision was made ~ 1 cm lateral to the midline on the symptomatic side. Sequential dilators were inserted to create a surgical pathway to the lumbar spine. It allowed placement of a 18-mm-diameter METRx tubular retractor with an endoscopic system (Medtronic, Langhorne, PA, USA) in groups A and C, while a 18-mm-diameter Zista tapered retractor (Bosscom Technology, Chongqing, China) (Figure 1) assisted with an operating microscope (Carl Zeiss, Inc., Oberkochen, Germany) was used in groups B and D. To maintain the position of the retractors, the tube was supported by an articulated metal arm that was attached to the operating table. A laminotomy was performed using a 6-mm diamond bur. In discectomy procedures, the herniated portion of the disk was removed in groups C and D and aggressive subtotal discectomy was never intended (Figure 2). Bilateral laminectomies were performed by moving the working tubular retractors medially and undermining the spinous process and contralateral lamina using a diamond bur and Kerrison rongeurs in 2 cases. Wound drainage was applied in all cases. Ambulation was permitted on the day of surgery.

### 2.3. Statistical Analyses

In this study, the statistical analysis was performed using SPSS software version 25.0 (IBM Corp, Armonk, NY). Values were demonstrated as the mean (SD) unless otherwise indicated. Student's t-test was used to assess the difference between the 2 groups at the same time points and a P-value <0.05 was considered statistically significant.

## 3. Results

### 3.1. Patients

All patients successfully underwent an endoscope-assisted or microscope-assisted tubular surgery for lumbar laminectomies or discectomies without conversion to open surgery. The mean age for patients with LSS (groups A and B) was 62.3 years, with a range of 30-83 years. The mean age for patients with LDH (groups C and D) was 47.5 years, with a range of 17-79 years (Table 1).

Data on operative times and intraoperative blood loss are given in Table 2. The results showed characteristic differences in operating times between endoscope-assisted and microscope-assisted procedures and between discectomies and laminectomies. The average operative time was 91.03 min in laminectomy groups (range: 45 min–200 min in group A and 50 min–134 min in group B), while it was 78.88 min in discectomy groups (range: 40 min–174 min in group C and 30 min–120 min in group D). The mean time of group A (95.23 min) was 9.10 minutes longer than the duration of group B (86.13 min), while the duration of group C (82.66 min) was 7.96 minutes longer than group D (74.70 min) (P < 0.05). The average blood loss was 41.86 ml and 38.5 ml in laminectomy groups, and 35.04 ml and 39.26 ml in discectomy groups, but the difference was not statistically significant. There was no difference in day of mobilization among the separate cohorts.

### 3.2. Surgery-Related Complications

Occurrence of intraoperative and postoperative complications was assessed in a separate group (Table 2). Dural tear was the most common complication. Intraoperative dural tears were observed in 8 patients (4.94%) who underwent endoscope-assisted surgery and in 2 (1.38%) who underwent microscope-assisted surgery. 1 patient (0.62%) from the endoscope group and 5 patients (3.45%) from the microscope group developed a superficial wound infection that required treatment with intravenous antibiotics. At 2 years, a total of 8 patients required a second operation for a recurrent disc herniation. The reoperation rate was 3.09% (n=5) after endoscope-assisted surgery and 2.07% (n=3) after microscope-assisted surgery.

### 3.3. Treatment Effects

**Figure 3:**
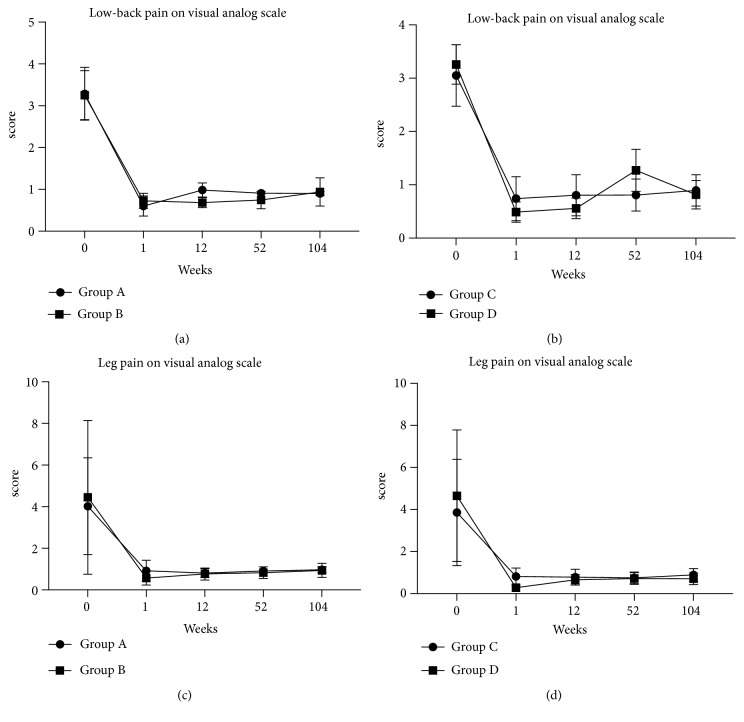
Curves of the mean scores on Visual Analog Scale (VAS) for low-back pain in laminectomy groups (a), VAS for low-back pain in discectomy groups (b), VAS for leg pain in laminectomy groups (c), VAS for leg pain in discectomy groups (d). Scores range from 0 to 10, with higher scores indicating more intense pain. VAS showed postoperative improvement in 4 graphs with a significant difference (P<0.01). The curves for the mean scores on the VAS did not differ significantly over the follow-up period of 2 years between groups.

Both tubular-based endoscope-assisted and microscope-assisted surgery were effective in relieving acute radicular symptoms. VAS scores of LBP and leg pain showed postoperative improvement in the four groups (P<0.01) (Figure 3). Follow-up examinations were conducted 3 months, 1 year, and 2 years after surgery. At the last follow-up, a significant and constant improvement was observed for VAS scores of LBP and leg pain, JOA scores, and ODI scores in all groups. The main difference between groups is that group B who underwent microscope-assisted tubular surgery reported better JOA recovery rate compared with group A treated with endoscope-assisted surgery (73.40% vs 51.19%, p < 0.05), while the difference was not statistically significant between groups C and D (Table 3).

## 4. Discussion

In recent years, the minimally invasive lumbar surgery has been developed and gained popularity for degenerative lumbar spinal disease. Laminectomies and discectomies are performed by using tubular retractors in conjunction with an endoscope or microscope to improve patient recovery while retaining surgical efficacy [6–9, 12]. In this study, the METRx tubular retractor with an endoscopic system was used in groups A and C, while the Zista tapered retractor with a microscope was used in groups B and D. The Zista tapered retractor is 22 mm in diameter on the upper end and tapers to a diameter of 18 mm at the opposite end. It allows more free movement and angulation of the surgical tools and the microscope than a cylindrical tubular retractor. This study revealed that the clinical outcomes for the endoscope-assisted and microscope-assisted tubular surgery were similar, while patients treated with the tubular microscope-assisted laminectomies were expected to have higher JOA recovery rate. It may be because the microscope could provide clearer three-dimensional (3D) vision with consequent complete decompression for lateral recess stenosis.

The most common complication was dural tear, which occurred more often in the endoscope-assisted group. Intraoperative dural tears were observed in 8 patients (4.94%) who underwent endoscope-assisted surgery and in 2 (1.38%) who underwent microscope-assisted surgery. Depth of field provided by 3D vision in microscope-assisted surgery may reduce an incidental dural tear, compared with two-dimensional imaging information in endoscope-assisted surgery.

Wound infection after tubular discectomy is uncommon [18], probably due to the smaller incision and minimized dead space. However, five patients (3.45%) developed a superficial wound infection in microscope-assisted group. The surgical microscope has been implicated as potential source of infection, even if the microscope is draped and sterile [19, 20]. Proper techniques of cleaning, storage, and draping should be used to minimize potential infection. We replaced gloves immediately after we adjust the microscope every time during the operation in the last 30 microscope cases, and no infection happened in these cases.

More surgical time was required in endoscope-assisted group C. The possible reason may be blood and bone detritus keeping splattering against the endoscope and blurring the camera lens during the drilling operation. During this procedure, surgeons might spend an additional time keeping the lens clean, compared with tubular-based microscope-assisted surgery.

However, the use of a microscope can be cumbersome during bilateral decompression via a unilateral approach because the microscope needs to maintain coaxial vision using the tubular retractor. The tubular retractor that is fixed to the endoscope allows convenient medial placement to view the contralateral side of the spinal canal. Tilting the operation table was unnecessary in both methods. In addition, in endoscope-assisted surgery, the surgeon does not have to worry about the working distance that the surgical microscope requires. These may be the reasons that some surgeons prefer to choose endoscope-assisted surgery over a microscope.

In discectomy procedures, the herniated portion of the disk was removed and aggressive subtotal discectomy was never intended. In a systematic literature review, aggressive discectomy is associated with an increased incidence of long-term recurrent back and leg pain but a lower incidence of recurrent disc herniation versus limited discectomy [21]. Studies support the hypothesis that increased disc disruption will accelerate degenerative disc disease [22, 23]. Furthermore, disc height collapse may lead to decreased foramen height, contributing to recurrent leg pain [21]. It is important to note that there were variations in the amount of disc removed in each case.

However, there were several limitations to this study. First, the retrospective nature of the study made it difficult to obtain reliable information on all patients. There is no randomized clinical study, and the decision on surgical strategy was based on the preferences of patients. Second, it was performed by one surgical team in a single institution, so the results could be biased. Multicenter studies with long-term follow-up data will be required to draw up a conclusion in the future. Finally, only patients with distinct herniated disks were included in LDH group, while patients with less distinct compression on MRI were excluded. It may be bias to assume that the study is not valid for these patients.

## 5. Conclusions

Both endoscope-assisted and microscope-assisted tubular surgery for laminectomies and discectomies of degenerative spinal disease are effective treatment modalities. This study provides evidence that tubular-based microscope-assisted surgery may relate to better recovery rate for LSS, less surgical time, and less intraoperative dural tear compared with endoscope-assisted tubular surgery.

## Figures and Tables

**Table 1:** Patient demographics.

	Group A (n=35)	Group B (n=30)	Group C (n=127)	Group D (n=115)
Age, mean (SD), year	61.66(13.07)	62.93(12.26)	48.69(13.07)	46.3(13.98)
Female sex, No.(%)	16(45.7)	14(46.7)	53(41.7)	52(45.2)
Levels				
L3-4	3	0	4	2
L4-5	26	27	75	58
L5-S1	6	3	48	55

**Table 2:** Operative characteristics of patients.

	Group A	Group B	Group C	Group D
Duration of operation, mean (SD), min	95.23(38.36)	86.13(26.04)	82.66(24.97)	74.70(20.23)*∗*
Amount of bleeding, mean (SD), ml	41.86(52.46)	38.5(23.24)	35.04(46.20)	39.26(20.81)
Hospital stay, mean (SD), day	5.5(2.9)	5.2(1.3)	4.4(1.7)	4.5(1.5)
Dural tear, n (%)	2(5.71)	0	6(4.72)	2(1.74)
Wound infection, n (%)	0	1(3.33)	1(0.79)	4(3.48)
Repeated surgery within 2 y, n (%)	1(2.85)	0	4(3.15)	3(2.61)

Values are presented as the mean (SD) unless otherwise indicated. *∗*P<0.05 in duration of operation (group C vs. group D). SD: standard deviation.

**Table 3:** Treatment effects (ODI and JOA).

	Group A	Group B	Group C	Group D
Initial ODI, mean (SD), %	53.95(17.46)	53.85(10.22)	49.42(17.56)	59.08(19.84)
104-week ODI, mean (SD), %	18.44(15.22)	11.18(15.15)	9.99(12.7)	11.72(11.69)
Initial JOA, mean (SD)	13.66(6.68)	13.30(5.45)	13.88(5.40)	11.37(6.72)
104-week JOA, mean (SD)	22.49(6.34)	25.62(4.03)	26.29(3.23)	26.29(2.91)
JOA recovery rate, mean (SD), %	51.19(44.89)	73.40(41.04)*∗*	80.83(25.16%)	81.60(21.42)

Oswestry Disability Index (ODI) scores and Japanese Orthopaedic Association (JOA) scores at admission and 104 weeks postoperatively in the 4 groups. The lowest ODI score corresponds to the best functional state. The JOA recovery rate was calculated according to the following formula: (104-week follow-up score - preoperative JOA score)/(29 - preoperative JOA score) × 100%. *∗*P<0.05 in JOA recovery rate (group A vs. group B).

## Data Availability

The data used to support the findings of this study are available from the corresponding author upon request.

## Abbreviations

LSS: Lumbar spinal stenosis
LDH: Lumbar disc herniation
VAS: Visual analogic scale
LBP: Low-back pain
ODI: Oswestry Disability Index
JOA: Japanese Orthopaedic Association
MD: Microdiscectomy
MED: Microendoscopic discectomy
MRI: Magnetic resonance imaging
CSF: Cerebrospinal fluid
Inf: Inferior
Lat: Lateral
Med: Medial
Sup: Superior
SD: Standard deviation.
